# Neural mechanisms of social dominance

**DOI:** 10.3389/fnins.2015.00154

**Published:** 2015-06-17

**Authors:** Noriya Watanabe, Miyuki Yamamoto

**Affiliations:** ^1^Research Fellow of the Japan Society for the Promotion of ScienceTokyo, Japan; ^2^Department of Psychology, Graduate School of Environmental Studies, Nagoya UniversityNagoya, Aichi, Japan; ^3^Center for Information and Neural Networks, National Institute of Information and Communications TechnologySuita, Osaka, Japan; ^4^Faculty of Human Sciences, University of TsukubaTsukuba, Ibaraki, Japan

**Keywords:** social hierarchy, amygdala, striatum, prefrontal cortex, parietal cortex, monoamine systems, NPB/W system

## Abstract

In a group setting, individuals' perceptions of their own level of dominance or of the dominance level of others, and the ability to adequately control their behavior based on these perceptions are crucial for living within a social environment. Recent advances in neural imaging and molecular technology have enabled researchers to investigate the neural substrates that support the perception of social dominance and the formation of a social hierarchy in humans. At the systems' level, recent studies showed that dominance perception is represented in broad brain regions which include the amygdala, hippocampus, striatum, and various cortical networks such as the prefrontal, and parietal cortices. Additionally, neurotransmitter systems such as the dopaminergic and serotonergic systems, modulate and are modulated by the formation of the social hierarchy in a group. While these monoamine systems have a wide distribution and multiple functions, it was recently found that the Neuropeptide B/W contributes to the perception of dominance and is present in neurons that have a limited projection primarily to the amygdala. The present review discusses the specific roles of these neural regions and neurotransmitter systems in the perception of dominance and in hierarchy formation.

## Introduction

The perception of social rank is a very important skill that must be exercised during daily human interactions. Whether at work, school, or home, humans consciously or unconsciously alter their attitudes by adapting themselves to the social status of others. The misinterpretation or ignorance of the social dominance ranking of an individual may lead to serious consequences, such as exclusion from a social group. Recently, the field of social neuroscience has begun to study the neural substrates that underlie social dominance and the formation of social hierarchies using a number of approaches, including a variety of animal models and brain imaging methods in humans.

The definition of social dominance varies according to the researcher. In the field of personality psychology, Schutz (1958) first described the human characteristics of dominance as one dimension of interpersonal personality using the term “control” which may be defined as the tendency to control or be controlled by others. Similarly other researchers described dominance as the motivation for control (Gough, 1975; Ellyson and Dovidio, 1985; Dépret and Fiske, 1993; Berger, 1994; Burgoon et al., 1998; Burgoon and Dunbar, 2000; Keltner et al., 2003). In these studies, dominance is defined as a personality trait which involves a motive to control others, the self-perception of oneself as controlling others, and/or a behavioral outcome resulting from these motives or perceptions (for a review, see Hall et al., 2005).

In the psychological field of emotion, dominance is included as a factor that defines emotion. For example, Mehrabian proposed a temperament model in which human emotion can be described using a three-dimensional model that includes Pleasure-Displeasure, Arousal-Calm, and Dominance-Submissiveness (the PAD theory; Mehrabian, 1972, 1996; Russell and Mehrabian, 1977). Moreover, he described dominance as one of the principle features used to evaluate one's own emotion. In the PAD theory, the Dominance-Submissiveness axis is defined as a feeling of control and influence over one's surroundings and others vs. a feeling of being controlled or influenced by one's surroundings and others. This definition is similar to that by personality psychology.

Thus, in the evaluation of personality traits and emotions, dominance is often associated with the concept of control. Therefore, the present review defines dominance as a mental state in which one feels that he/she is superior to and in control of others, or is inferior to and under the control by others. This definition can be applied if a subject compares two people's relative ranks based on the observation which is superior to and in control of the other. The definition can also be extended to non-human animal by observing specific behaviors such as the expression of aggression or submissiveness, or ranking of food access (Bekoff, 1977; Zumpe and Michael, 1986; Santos et al., 2012).

## Social hierarchy and dominance

Social hierarchy is a form of the expression of dominance that is observed in a variety of animal species that develop communal systems, from fish to primates (Paz-Y-Mino et al., 2004; Grosenick et al., 2007; Byrne and Bates, 2010). Several aspects of behavior, including food acquisition and breeding, are influenced by social hierarchy and, in fact, some species exhibit morphological changes according to their hierarchical rank within a society. For instance, flanges (cheek-pads) appear on the face of a male orangutan only when that individual is physically strong and socially dominant (Mackinnon, 1974; Kuze et al., 2005). However, social rank-induced changes are not limited to physical appearance, and a number of social signals related to dominance influence the activity of brain systems (Sapolsky, 2005).

Human social systems have also evolved based on social hierarchy, which have emerged to increase the probability of survival in hazardous situations. If a group functions as well as, and similar to, a single organic system, then that group can achieve far more than a lone individual. For this to occur, individuals are generally required to function under a single control center and a component of hierarchical information processing. In animal societies, physical strength tends to determine social rank but in human societies it is not only physical strength but also cognitive factors such as intelligence and emotional stability that determine his/her social ranking (Hall et al., 2005). In humans, recent study (Cook et al., 2014) reported that there are two types in dominant personalities; one they named social dominance and the other aggressive dominance. The former rely on persuading others by reasoning, and the latter uses aggression, threat, deceit and flattery. Although strategies are different, both types have a motivation to control others and understand their hierarchical relationships for the control. In human children, the concept of dominance develops at around the age of 10 months, which is prior to language acquisition, and children of that age can distinguish the dominance of two agents based on body size (Thomsen et al., 2011). At the age of 15 months, children can infer whether an individual is dominant or not based on their previous subjective experiences (Mascaro and Csibra, 2012). Thus, in a human society, the dominance is perceived by a simple physical factor such as the body size, however, the learning experiences based on interactions with other individuals, or on observation of other individuals' interactions within a social framework seem to be incorporated into the conceptual formation of dominance and a social hierarchy. Furthermore, humans learn that a social dominance hierarchy is a set of implicit social norms that guide behavior according to social status (Cummins, 2000).

Recently, the neural substrates underlying the perception of social dominance have been studied in humans using functional magnetic resonance imaging (fMRI). In the present review, the neural structures and learning processes that are involved in the perception of dominance in a group setting mainly by this method, and mechanism that may work for the maintenance of dominant position after social hierarchy formation are summarized.

## Facial expression and dominance

During direct (face-to-face) communication, an individual can perceive the social status or hierarchical rank of other individuals in a social group through various clues. An individual tends to alter their behavior based on the relative social rank of the other compared to his/her own rank. One clue that may aid in the judgment of another individual's social rank is facial expressions.

Wiggins proposed the interpersonal circumplex model with two-axis concept of Valence and Dominance/Power for the evaluation of interpersonal behavior. (Wiggins, 1979; Wiggins et al., 1989). Results of Oosterhof and Todorov (2008) supported Wiggins' model. They examined the impressions of participants during the observation of a variety of human faces. To avoid the emotional component inherent in facial expressions, they used photographs of neutral faces with no clear emotional expression. The participants were asked to describe their impressions of the neutral faces on a scale from 1 to 9 using 15 adjective rating measures that included terms such as “attractive,” “weird,” “mean,” and “trustworthy” and they identified independent facial features using principle component analysis. Two orthogonal (independent) axes were extracted: Valence and Dominance/Power. Oosterhof and Todorov concluded that people typically evaluate the faces of others based on whether they appear favorable (Valence axis: high scores of trustworthiness, emotionally stable, and responsible) or whether the person is dominant (superior) to the participant (Dominance/Power axis: high scores for dominant, confident, and aggressive). Thus, they suggested that one of factors that determines interpersonal relationship is Dominance/Power.

**Figure 1:**
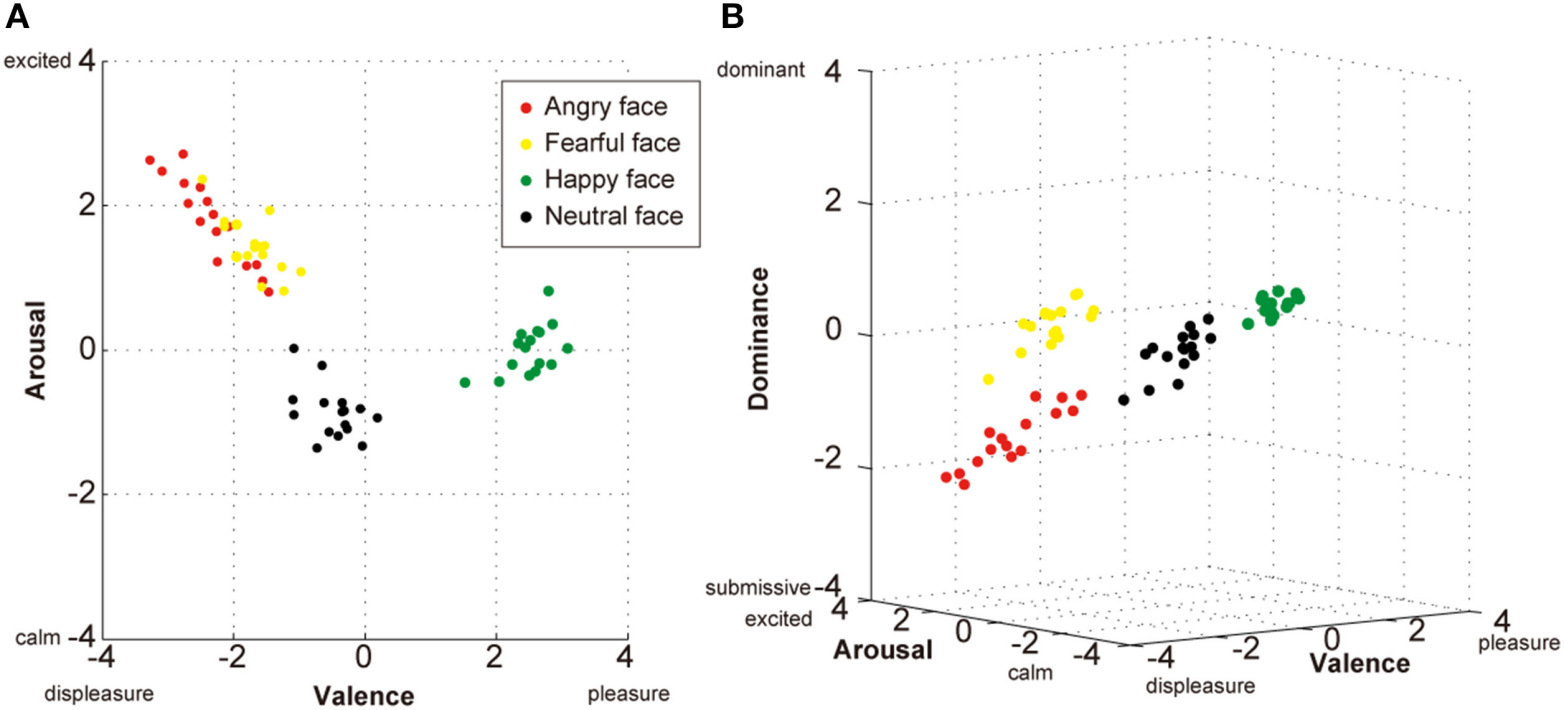
**Two- and three-dimensional plots of affective space for the evaluation of facial expressions. (A)** Two-dimensional plots (Valence and Arousal) based on the circumplex model of subjective emotion (Russell, 1980) demonstrating poor discrimination during the self-evaluation of angry (red dots) and fearful (yellow dots) faces. **(B)** Three-dimensional plots (Valence, Arousal, and Dominance) based on the Pleasure–Displeasure, Arousal–Calm, and Dominance–Submissiveness (PAD) model (Russell and Mehrabian, 1977; Mehrabian, 1996) demonstrating a better recognizable discrimination of all four facial expressions. Each plot shows the average evaluation (*n* = 122 participants) of each stimulus (16 stimuli × four expressions). Watanabe et al. (2012) investigated the effect of a single nucleotide polymorphism (SNP) in Neuropeptide B/W receptor 1 (NPBWR1); however, the present data plots disregarded the different SNP types to better describe the general tendencies of perceived emotion from the four types of facial expression.

Similarly in the field of psychology of emotion, Russell and Mehrabian (1977) proposed a three-dimensional theory which is defined by the axes of Valence, Arousal, and Dominance. However, Russell (1980) later removed the Dominance axis and defined emotion using only the Valence and Arousal axes in his “Circumplex model.” Recently, using pictures of emotional faces to evaluate the evoked emotional state of the observer, Watanabe et al. (2012) found that the three-dimensional model of Valence-Arousal-Dominance provided a better explanation of the observers' emotional perception of faces than the two-dimensional Valence-Arousal model. In this experiment, the participants were presented with four types of emotional faces that were classified into four categories: angry, fearful, happy, and neutral (from NimStim face stimulus set by Tottenham et al., 2009). The Self-Assessment Manikin Scale (Bradley and Lang, 1994), which is based on the three-factor theory of Russell and Mehrabian (1977; see also Mehrabian, 1996), was used to assess emotions experienced by participants. They rated each picture according to the intensity of their emotional reaction for each of the three scales (Valence, Arousal, and Dominance) on a nine-point scale (from −4 to +4 with 0 as a neutral point). After plotting all of the ratings in either a two-dimensional or three-dimensional space, a discrimination analysis was used to determine whether each stimulus could be differentially reclassified into one of the original four categories (Figure 1).

When the evaluation scores were plotted using the two-axis model (Valence-Arousal), the happy and neutral faces were discriminated with 100% accuracy but 25% of the angry faces were misclassified as fearful faces and 18.8% of the fearful faces were misclassified as angry faces (Figure 1A). In contrast, when the three-dimension model (Valence-Arousal-Dominance) was used, all stimuli fell into four separate clusters and the angry and fearful faces as well as happy and neutral faces were each discriminated with 100% accuracy (Figure 1B). Thus, when an individual encounters an angry or fearful face, the Valence and Arousal assessments may be similar because both types of stimuli are alarming and not readily likeable. However, if the Dominance axis is included in the assessment, then the angry faces are clearly differentiated and described as intimidating while fearful faces do not evoke a feeling of intimidation. Thus, it seems more appropriate to include the Dominance dimension when evaluating the emotional reaction of an individual to human faces. These results indicate that one of factors that people use for evaluating their own social ranks is others' facial expressions, and suggested that brain areas that are involved in emotional information processing of face such as amygdala may also play important roles in the perception of dominance.

In the following sections, the manner in which the perception of dominance is coded in various brain regions, particularly the cortical and subcortical systems (Section Neural Substrates of Social Dominance), and how neurotransmitter systems influence the formation and maintenance of a social hierarchy (Section Neurotransmitters Involved in social dominance and hierarchy formation) are described.

## Neural substrates of social dominance

In the last decade, researchers in the field of neuroscience have attempted to decipher the neural mechanisms that support behaviors in the social domain (review by Singer, 2012). For example, the brain regions that are activated when an individual assesses the hierarchical relationship between him/herself and another individual or two other individuals have been studied in various contexts (see Table 1). It is important to note that the experimental stimuli and/or behavioral measurements used in each study vary and, as a result, the neural activation patterns observed among these studies tend to differ based on the stimulus parameters and experimental conditions. However, these studies have consistently identified several brain regions as involved in the perception and learning of social dominance, including the amygdala, the hippocampus, the striatum, the intraparietal sulcus (IPS), the ventromedial prefrontal cortex (VMPFC), and the lateral prefrontal cortex (LPFC).

**Table 1:** **Summary of the brain regions related to social dominance**.

**Region**	**Region (Details)**	**Animal**	**Methods**	**Contexts**	**Reported features**	**Article**
Amygdala					**Lesion effects**	
	Including temporal lobe	Monkey	Lesion	Social	• Lost their social status in the group	Rosvold et al., 1954
	–	Monkey	Lesion	Social	• Insensitivity to social threats	Machado and Bachevalier, 2006
	–	Monkey	Lesion	Social	• Insensitivity to social threats	Emery et al., 2001
	–	Human	Lesion	Social	• Abnormal inter-individual distance	Kennedy et al., 2009
					**Ranking dependent brain activities**	
	–	Human	fMRI	Social (unstable)	• Superior > inferior human player	Zink et al., 2008
					• Correlated with the individual motivation to be the top rank	
					**Learning of hierarchy**	
	–	Human	fMRI	Social vs. Non-social	• Confidence level of human hierarchy learning	Kumaran et al., 2012
			fMRI	Social vs. Non-social	• Correlated with social rank in invest game	
			Morphometry	Social vs. Non-social	• Learning performance of human hierarchy ranking is correlated	
					**Ranking dependent individual difference**	
	–	Monkey	Morphometry	Social	Correlated with social dominance ranking	Noonan et al., 2014
Hippocampus					**Learning of hierarchy**	
	Anterior	Human	fMRI	Social vs. Non-social	• Correlated with confidence level of human hierarchy learning	Kumaran et al., 2012
	Posterior			General	• Correlated with confidence level of general hierarchy learning	
					**Ranking dependent brain activities**	
	Parahippocampus	Human	fMRI	General	• Superior > inferior human and computer player	Zink et al., 2008
Striatum					**Ranking dependent brain activities**	
	Ventral	Human	fMRI	General	• Superior > inferior human and computer player	Zink et al., 2008
	Dorsal		fMRI	Social vs. Non-social	• Defeating > defeated by superior human player	
	Ventral	Human	fMRI	Social	• High activity to similar social status person	Ly et al., 2011
IPS					**Ranking dependent brain activities**	
	LIP	Monkey	Single unit recording	Social	• Superior > inferior monkey face	Klein et al., 2008
	Including parietal and occipital area	Human	fMRI	General	• Superior > inferior human face in stable hierarchy condition	Zink et al., 2008
	–	Human	fMRI	General	• Distance (magnitude) ranking comparison	Chiao et al., 2009
VMPFC					**Lesion effects**	
	–	Human	Lesion	Social	• Become less sensitive to specific social dominance cues	Karafin et al., 2004
					**Learning of hierarchy**	
	–	Human	fMRI	General	• Correlation with confidence level of general hierarchy learning	Kumaran et al., 2012
LPFC					**Ranking dependent brain activities**	
	DLPFC (BA9,46)	Human	fMRI	Social	• Superior > inferior human face	Zink et al., 2008
	DLPFC(BA46), VLPFC(BA47)	Human	fMRI	Social	• Dominant > equal or submissive body language	Marsh et al., 2009
					**Context dependent brain activities**	
	DLPFC (BA9,46)	Human	fMRI	Social vs. Non-social	• Human > computer player	Zink et al., 2008
	VLPFC(BA47)	Human	fMRI	Social vs. Non-social	• Social status > digit magnitude (non-social)	Farrow et al., 2011

The term “Social” refers to a statistically significant result in terms of social contexts without a direct comparison to non-social contexts. The term “General” refers to a statistically significant result in terms of both social and non-social contexts. The term “Social vs. Non-social” refers to a result which showed significant difference between social and non-social contexts.

### The amygdala

The amygdala is generally considered as the center of emotional responsiveness (Ledoux, 2007). Additionally, this brain region has high sensitivity to social information such as trustworthiness and social rewards (Adolphs, 2010). The first study to investigate the function of the amygdala in terms of the social behavior of non-human primates (Rosvold et al., 1954) found that high-ranking monkeys with surgical lesions of the amygdala lost their status in the social dominance hierarchy and became extremely submissive. Later studies showed that monkeys with selective bilateral lesions of the amygdala were insensitive to threatening social signals (Machado and Bachevalier, 2006) and had shorter contact latencies with novel monkeys than did controls (Emery et al., 2001).

Consistent with findings from primates, a human patient with bilateral lesions of the amygdala (Urbach-Wiethe disease) exhibited inappropriate social judgments (in terms of approachability and the trustworthiness of unfamiliar individuals) and failed to maintain employment throughout her life (Adolphs et al., 1995, 1998). In humans, interpersonal distance is one recognizable measure of non-verbal social dominance (Hall et al., 2005). Likewise, patients with amygdala damage tend to lack any sense of interpersonal distance. For example, the measured comfortable interpersonal distance of a patient with amygdala lesion was 0.34 m whereas that of controls was 0.76 m (Kennedy et al., 2009). Additionally, fMRI results from that study revealed that the blood-oxygen-level dependent (BOLD) signal from the amygdala of healthy controls increased when the participant knew an experimenter was close to the scanner (and, thus, to him/her) compared to when the experimenter was far from the scanner. These findings suggest that the amygdala is also involved in sensing the interpersonal distance, which is an indicator of social dominance perception in terms of territory.

Activity in the amygdala can also be modulated by factors such as the nature of a hierarchy (stable or unstable) or the context of a ranking (social or unsocial). Zink et al. (2008) investigated dominance-related brain activity using virtual game rankings that were indicated by stars near the face of each player. Each participant was assigned to the middle rank and required to win the game when he/she played against either a superior or inferior player under two conditions: the stable hierarchy condition in which the ranking of the participant did not change and the unstable hierarchy condition in which the ranking of they could move up or down according to the result of the game. As results, only during the unstable hierarchy game, the amygdala was activated to a greater degree by stimuli associated with superior-ranked players than by those associated with inferior-ranked ones (Zink et al., 2008). Furthermore, changes in the BOLD signal in the amygdala were correlated with the participant's subjective ratings of their positive feelings following a win against a superior player.

There is also one evidence demonstrating the involvement of the amygdala during the inference of social ranking. Kumaran et al. (2012) investigated the learning processes associated with social hierarchy and the related alterations in brain activity using fMRI. They introduced “inference score index” as a proxy for the evaluation of the level of hierarchical knowledge. During the learning session of the experiment, their participants were required to learn the hierarchical structure of the social ranks of people in a group, and galaxy ranks depending on mineral content which represents non-social rank as a scientific fiction story. As correctness of their answers were feed-backed, they could learn the ranking of the person or galaxy in a gradual manner. In the testing session, the participants were required to determine the hierarchical rank of two people and indicate their level of confidence in their answer using a scale from 1 (guess) to 3 (very sure); the inference score index was calculated by multiplying the correctness of the response (0 or 1) with subjective the confidence rating (1, 2, or 3). As the learning session progressed, the inference scores of them increased and, thus, the inference score index could be used as a proxy for the level of hierarchical knowledge attained by a subject during the learning phase. They found that bilateral activation of the amygdala (and the anterior hippocampi) was correlated with the confidence level of the social ranking inferences, but not the non-social ranking inferences. After learning both the social and non-social rankings, the participants engaged in two types of game; “bid trial” game and “control trial” game. In the bid trials, they were required to use their knowledge about the person (social) and the galaxy (non-social) hierarchies to decide how much money to invest in potential projects whose success probabilities depended solely on the sum of both of these ranks. In this situation, higher rankings in each category were associated with greater participants' motivation. During the investing phase of this game, activation of the amygdala was correlated only with social ranking, whereas VMPFC and posterior hippocampal activation were positively correlated with both social and non-social rankings. However, in the “control trials” in which they simply compared the both categories of the stimuli without making an investment, there is no significant correlation between non-social rank and the amygdala activation. These findings suggest that social ranking information encoded in the amygdala is modulated by motivational inputs (amount of rewards). This notion is consistent with the findings of Zink et al. (2008) because in that study the amygdala was activated only when the participants had a strong motivation to win the game and had the opportunity to be a superior player. Thus, activity in the amygdala likely represents the learning processes that are specific to determining a social hierarchy and can be modulated by motivational input.

In addition to these activity change during the perception of ranking, morphological difference by voxel-based morphometry (VBM) also showed relationship between the amygdala and social dominance. Kumaran et al. (2012) investigated the relationship between the learning of a social hierarchy and the morphological features of the amygdala. They found that individual differences in gray matter volume in the amygdala were correlated with social inference performance such that a higher inference score was associated with a larger amygdala volume. Similar morphological difference in amygdala was observed in macaque monkeys. Noonan et al. (2014) reported that individual social status in the group were positively correlated with their amygdala size. Thus, the amygdala seems to be involved in the formation and maintenance of a social hierarchy as well as the perception and learning of social dominance.

### The hippocampus

Kumaran et al. (2012) also described the differential roles of the anterior and posterior hippocampi during social and non-social ranking tasks in conjunction with amygdala-specific activity that was associated with the level of confidence of subjective inferences regarding social rank. Activation of the anterior hippocampus, which has strong anatomical connections with the amygdala (Aggleton, 1986; Saunders et al., 1988), was correlated with individual level of confidence in their inferences of social, but not non-social, rankings while posterior hippocampal activity was correlated with that of both social and non-social rankings. Similarly, Zink et al. (2008) found that activity in the parahippocampal cortex, the reported coordinates of which were similar to those of the posterior hippocampus in Kumaran et al. (2012), was modulated in both social and non-social contexts.

### The striatum

The striatum codes value, saliency, and reward-prediction-error signals (Schultz et al., 1992; Tremblay et al., 1998; Breiter et al., 2001; Knutson et al., 2001; McClure et al., 2003; O'Doherty et al., 2003, 2004; Samejima et al., 2005; Matsumoto and Hikosaka, 2009). Zink et al. (2008) used a simple reaction time task to assess the role of the striatum during the perception of dominance. As described in Section The Amygdala, in their experiment, participants competed in terms of reaction speed with other players, whose pictures were displayed together with their ranking indicators. The fMRI findings of Zink et al. (2008) show that viewing the face of a higher-ranked opponent elicited a greater degree of activity in the ventral striatum than when viewing the face of a lower-ranked opponent, regardless of whether this was in a social (vs. a human player) or non-social (vs. a computer player) context. The authors concluded that activation of the ventral striatum is derived from the assignment of a greater value or salience to a higher-status player. They also found that the striatum was activated to the greatest degree when participants were informed of their win or loss and when they defeated a superior human player (social context). However, this activation did not occur when participants defeated a superior computer player (non-social context). This raised the question of how such a specific type of striatal activation was elicited by social context. Generally, people are highly sensitive to rewards in competitive social situations (Fliessbach et al., 2007; Bault et al., 2011) and, accordingly, the participants in Zink et al. (2008) reported a greater motivation to win when playing a human player compared to a computer. Thus, context-dependent activity in the striatum may reflect motivational differences when an individual is competing against a human rather than a computer, and against a higher-ranked opponent rather than a lower-ranked one.

Striatal activity is also affected by the subjective sensitivity of a participant to gains and losses and by their current emotional state (Tom et al., 2007; Delgado et al., 2008; Watanabe et al., 2013). Consistent with this notion, Ly et al. (2011) found that striatal activation is dependent on the subjective social status of a participant based on socioeconomic rank and the statuses of other people according to the MacArthur Scale of Subjective Social Status (Adler et al., 2000). Their fMRI results revealed that striatal activity was dependent on the interaction of the individual status and that of the stimulus such that high-status individuals exhibited a greater striatal response to high-status information and low-status individuals exhibited a greater striatal response to low-status information. Thus, striatal activity may code social ranking based on a skewed sensitivity, which peaks around the hierarchical status of the participant.

### The intraparietal sulcus (IPS)

In primates, the perception of dominance as it is related to attentional orienting seems to be associated with the IPS. A behavioral study of male rhesus macaques found that visual orienting decisions were influenced by the social status of a particular stimulus (Deaner et al., 2005). In the study, the monkeys performed a visual-choice task in which gaze-shifting to one target (T1) delivered only juice whereas gaze-shifting to another target (T2) delivered juice as well as the display of an image, which was the familiar face of either a superior or inferior monkey. The substitutability of the image and the fluid rewards were estimated by varying the amount of juice that was delivered following the choice of either T1 or T2. The findings show that the monkeys allocated a higher value to watching superior monkey images than inferior monkey images. Electrophysiological evidence supporting these behavioral findings was later observed in the lateral intraparietal area (LIP), which is the lateral inferior aspect of the IPS in macaque monkeys (Klein et al., 2008). These authors found that neurons in the LIP exhibited higher firing rate when subjects chose the face of a superior monkey compared to the face of an inferior monkey. Interestingly, there was no modulation of the firing rate when a single target was presented and no choice was necessary. These data demonstrate that LIP neurons represent value within a social hierarchy during the active decision-making of a monkey.

Similarly, although there is some disagreement regarding the topological and functional homologies of the monkey IPS (Culham and Kanwisher, 2001; Mars et al., 2011), several fMRI studies of humans also have identified the involvement of the IPS during the perception of dominance. Activity in the bilateral occipital and parietal cortices is significantly greater when participants view a superior player compared to an inferior player when there is no change in hierarchy (Zink et al., 2008). It is known that IPS is involved during magnitude judgments, such as in a number-comparison task that requires participants to judge which of a pair of digits is larger (Dehaene et al., 2003). In that fMRI experiment, the IPS exhibited a greater degree of activation (and a longer reaction time) during the comparison of a number pair with a close distance than during a number pair with a far distance (and a shorter reaction time). Chiao et al. (2009) hypothesized that this IPS-mediated magnitude effect would be observed not only during the comparison of numbers but also during the comparison of social hierarchy relationships. Their study revealed that IPS activity was modulated by social status indicators such as cars, the medals of military officers or the face of the officers. Furthermore, a greater degree of activity was observed in the IPS when the hierarchical difference between two stimuli was close than when the difference was far. Thus, in IPS, information of “rank” regardless its content (social or non-social) might be processed in the similar way as information processing of “magnitude.”

### The ventromedial prefrontal cortex (VMPFC)

Some studies have indicated that the VMPFC may play a specific role for perceiving dominant cues (Karafin et al., 2004; Marsh et al., 2009). For example, patients with VMPFC lesions treated the head of the department, a postdoctoral student, and an undergraduate summer intern at a hospital equally, which suggests that these patients were relatively inattentive to social hierarchy cues (Karafin et al., 2004). These patients (*n* = 15) were also asked to evaluate social dominance based on pictures of faces but their mean dominance ratings did not differ from those of a control group. However, the standard deviation of the ratings was significantly smaller in the VMPFC-lesion group than in the control group. The authors of the study suggested that, rather than being incapable of making social dominance judgments, the patients with VMPFC lesions were less sensitive to the social value of specific perceptual cues such as age and gender.

In Kumaran's experiment (2012), the inference score index for both the social and non-social rankings (see Section The Amygdala for detail) were correlated with the activity in the VMPFC. However, specific roles of VMPFC in dominance perception still need to be clarified.

### The lateral prefrontal cortex (LPFC)

LPFC has been shown to play an important role in the perception of “social” dominance. Zink et al. (2008) investigated social dominance related brain activity using virtual game rankings with stable and unstable contexts (see Section The Amygdala for detail). In both conditions, there was a significantly stronger activation of the dorsolateral prefrontal cortex (DLPFC; Brodmann Area [BA] 9 and 46) when the participants observed the face of a higher-ranking player compared to when they observed the face of a lower-ranking player.

In a similar study, Marsh et al. (2009) measured brain activity in response to non-verbal stimuli (brow position, open–closed posture, direct–indirect gaze, and outwardly–inwardly gesture) that were indicative of the dominance level of an individual (dominant, equal, or submissive to the participant) in a picture. The DLPFC (BA 46) and the ventrolateral prefrontal cortex (VLPFC; BA 47) exhibited higher activation in response to a picture with a posture that reflected high social dominance compared to those showing equal or lower social dominance.

Both of these experiments indicate that the observation of a relatively dominant human induces a greater degree of activity in the lateral prefrontal cortices. Interestingly, Zink et al. (2008) also found that the social rank-induced differences in brain activation disappeared when their participants were informed that the superior/dominant player was a computer and not a real human. This implies that rank-associated differences in lateral prefrontal activity are specific to human social hierarchies. A similar specificity of activation to social hierarchy by the VLPFC (BA 47) was observed by Farrow et al. (2011). In this study, the VLPFC showed higher activity when their participants were asked to compare the social status of people in pictures than when they were asked to compare the magnitude of digits.

The manner in which this specificity emerges in the LPFC is unknown but the attentional system may be partly associated with this phenomenon. Several reports have found that the LPFC is involved in the attention systems of both humans (Desimone and Duncan, 1995; Miller and Cohen, 2001) and monkeys (Emery, 2000; Deaner et al., 2005) and that more attention is paid to hierarchically superior persons (or monkeys) than to inferior ones. In contrast, a non-social context may not induce this large degree of modulation of attentional intensity based on hierarchical differences. The greater activity observed in the LPFC during social interaction with socially dominant persons might reflect the intensity of attention.

An alternative explanation is that the LPFC processes information that is specific to social situations. For example, Spitzer et al. (2007) found that the LPFC (especially the right DLPFC) played an important role in social norm compliance during the performance of a game in which a participant could distribute money units freely to others under two conditions: a control condition in which there was no punishment if they behaved unfairly, and a punishment condition in which the subject could lose money as a punishment if they behaved unfairly. In this task, there was a greater degree of activation in the right DLPFC (BA 9 and 46) in the punishment condition compared to the control condition but this difference disappeared when the participants were instructed that the other player was a computer. Ruff et al. (2013) showed that social norm compliance levels could be modulated when transcranial direct current stimulation (tDCS) was applied to the right LPFC. This technique was effective in social contexts but not in non-social contexts. Thus, social norms may be coded in the LPFC and, because social hierarchy is one aspect of social norms (Cummins, 2000), the signals to enhance normative behavior may increase when exposed to a hierarchically dominant person.

Although these findings support the involvement of both the DLPFC (Zink et al., 2008; Marsh et al., 2009) and VLPFC (Chiao et al., 2009; Marsh et al., 2009; Farrow et al., 2011) in the perception of dominance, the functional differences between the VLPFC and DLPFC remain slightly confusing. This may be due to inconsistencies in the definitions of the DLPFC and the VLPFC or to the fact that a variety of experimental tasks were employed from study to study and, as a result, a direct comparison of these regions is not possible. Accordingly, the DLPFC and VLPFC likely engage in different cognitive demands (Hon et al., 2012; for review Duncan and Owen, 2000; Elliott, 2003). Regardless, in terms of social dominance, further studies that directly compare the roles of the dorsal and ventral prefrontal regions are needed.

### Summary of neural substrates of social dominance

These findings suggest that various brain regions are involved in the perception of dominance, and that these areas can be classified into two groups: one group that codes only social ranking and includes the LPFC, amygdala, and anterior hippocampus, and a second group that codes both social and non-social rankings and includes the VMPFC, IPS, striatum, and posterior hippocampus (Table 1). Amygdala was suggested to play an important role for the learning of social ranking. Striatum seems to process information of both social and non-social ranking in relation to value and reward system. IPS seems to code both types of ranking in relation to the “magnitude” and LPFC may code social ranking as a part of social norm. However, these notions are just beginning to be explored and future experiments will clarify roles of each brain regions in dominance perception.

## Neurotransmitters involved in social dominance and hierarchy formation

The following section summarizes the influence of the neurotransmitters involved in the perception of social dominance and the formation of a social hierarchy. The 5-HT and dopamine systems project throughout broad regions of the brain and regulate a variety of functions during the formation of a social hierarchy. Similarly, oxytocin levels throughout the brain are influenced by an individual's status within a hierarchy. In contrast, the recently discovered Neuropeptide B/W and its receptor NPBWR1 are also involved in the perception of social dominance but exhibit a very limited distribution in the brain (Table 2).

**Table 2:** **Neurotransmitters and hormones that influence social dominance**.

	**Animal**	**Neural origin**	**Target region**	**Effects**	**Article**
5-HT	Male vervet monkey	Raphe nucleus	Unknown	• Social hierarchy conditions increase serum 5-HT levels in dominant individuals	Raleigh et al., 1984
	Male vervet monkey			• Bidirectional modulation: blood 5-HT levels affect social hierarchy and social ranks affect blood 5HT levels	Raleigh et al., 1991
	Human			• Tryptophan administration enhanced dominant behavior	Moskowitz et al., 2001
Dopamine	Cynomolgus monkey	VTA, SNc	Striatum	• D2R expression increased in the striatum in dominant individuals	Grant et al., 1998; Morgan et al., 2002
	Human			• Subjective social status correlated with the D2R or D3R expression level (higher status with higher expression)	Martinez et al., 2010
Oxytocin	Female rhesus macaque monkey	Hypothalamic area	Unknown	• Serum oxytocin concentrations are higher in dominant females	Michopoulos et al., 2011
	Rat		Amygdala	• mRNA expression levels in the medial nucleus of amygdala were lower in subordinate rats	Timmer et al., 2011
NPB/W	Mouse	Hypothalamic area, Midbrain, and Pons	Amygdala, Hippocampus	• NPBWR1 KO mice showed abnormal contacts to the intruder	Nagata-Kuroiwa et al., 2011
	Human			• NPBWR1 SNPs showed different levels of dominance perception of human emotional faces.	Watanabe et al., 2012

5-HT, serotonin; VTA, ventral tegmental area; SNc, substantia nigra pars compacta; D2R, dopamine 2 receptor; D3R, dopamine 3 receptor; NPB/W, Neuropeptide B/W; NPBWR1, Neuropeptide B/W receptor 1.

Several endocrine systems also affects behavior and recognition of social dominance. As the influence of testosterone (Eisenegger et al., 2011; McCall and Singer, 2012) and corticosteroids (Sapolsky, 2005) on social dominance have already been extensively discussed in several reviews, we did not include these topics in this review.

### 5-HT system

Several studies have shown that this 5-HT system contributes to the formation of social hierarchy. Using measurements of 5-HT obtained from peripheral blood collected from the femoral veins of adult male vervet monkeys housed in groups, Raleigh et al. (1984) found that 5-HT levels depended on the social rank of a monkey, such that dominant monkeys had approximately twice the 5-HT concentrations of subordinate monkeys. However, the 5-HT levels of dominant monkeys were very sensitive to the presence of subordinates. When a dominant monkey was temporarily isolated, its 5-HT levels diminished to approximately the same level as those of the subordinate monkeys within 1 day. When these dominant monkeys were placed back into group housing, their 5-HT levels increased. On the other hand, the transition from a subordinate to a dominant position in the social hierarchy was accompanied by an increase in 5-HT levels. Unfortunately, because this study did not directly measure 5-HT levels in the brain, it cannot be determined whether these changes in social hierarchy were accompanied by changes in the neurobiological 5-HT system.

Raleigh et al. (1991) also examined whether 5-HT levels promoted the acquisition of dominance in adult male vervet monkeys by observing the hierarchical reshaping of a group. After the removal of the most dominant monkey from a group, certain subordinate monkeys were administered either tryptophan, a precursor of 5-HT (Young and Teff, 1989), to increase blood 5-HT levels, or fluoxetine, a selective 5-HT reuptake inhibitor (Gonzalez-Heydrich and Peroutka, 1990; Wong et al., 1990), to increase synaptic concentrations of 5-HT for 4 weeks. Compared with the non-treated controls in their group, subordinate monkeys who were treated with either tryptophan or fluoxetine exhibited greater levels of dominance within 4 weeks. Conversely, when the subordinate monkeys were administered fenfluramine, which disrupts 5-HT vesicle function when used in a chronic manner (Appel et al., 1990), or cyproheptadine, a 5-HT2A-receptor antagonist (Peroutka, 1988), the monkeys that received treatment forfeited their dominance and submitted to the non-treated controls within the group. These findings indicate that social dominance modulates internal 5-HT levels and that 5-HT levels can modulate vervet monkey hierarchy. Interestingly, Noonan et al. (2014) reported that the size of the raphe nucleus, which is the origin of 5-HT projection neurons (Hensler, 2006), is larger in dominant rhesus macaque monkeys than in subordinate monkeys. Although the study did not directly measure 5-HT levels in the brain, this observation is consistent with the idea that the 5-HT system influences the formation and maintenance of a social hierarchy.

Administration of 5-HT to humans has a similar effect on social dominance (Moskowitz et al., 2001). Healthy human participants received a dose of tryptophan (3 g/day) with their meals for 12 days and were asked to verbally describe their own communication frequency, agreeableness, and dominance. The participants who had been administered tryptophan exhibited an increase in dominant behavior and a decrease in quarrelsome behavior (critical comments of others).

### Dopaminergic system

Stress results in increased synaptic dopamine levels in the midbrain and chronic stress causes a downregulation of dopamine D2 receptors (D2Rs; Cabib and Puglisi-Allegra, 1996). In a positron emission tomography (PET) study of social hierarchy, dominant cynomolgus monkeys had greater binding of the D2R ligand [18F]fluoroclebopride ([^18^F]FCP), which has high affinity for D2Rs in the basal ganglia, than did subordinate monkeys (Grant et al., 1998). Because the binding affinity of a ligand is typically directly proportional to the number of D2R binding sites (Mach et al., 1996), these findings indicate that the chronic stress experienced by subordinate monkeys causes downregulation of D2R expression. However, that study did not directly determine whether this difference was the result of a decreased number of D2Rs in subordinate monkeys or an increased number of D2Rs in dominant monkeys. Moreover, it was also unclear whether the differential expression of D2Rs reflected a neurobiological predisposition that predetermined hierarchical rank or a neurobiological alteration that was induced by the attainment of a particular hierarchical rank.

A comparison of D2R levels among individual- and group-housed cynomolgus monkeys revealed that, rather than D2R levels predetermining social rank, the formation of a social hierarchy produced a D2R gradient (Morgan et al., 2002). Furthermore, compared to pre-individual housing, the binding of [^18^F]FCP increased in all monkeys after they were housed together, such that the most dominant monkey exhibited a greater degree of binding than did the subordinate monkeys. Thus, although Grant et al. (1998) concluded that rank-dependent differences in the binding of FCP are the result of D2R downregulation in subordinate monkeys experiencing chronic stress, it is more likely that these differences are the result of increased D2R binding in dominant monkeys (Morgan et al., 2002).

Similar effects were reported in a human study that used the Barratt Simplified Measure of Social Status (BSMSS) to evaluate social status and PET scans with [^11^C]raclopride to assess D2R and D3R binding in the striatum (Martinez et al., 2010). BSMSS scores were positively correlated with the level of [^11^C]raclopride binding, which supported previous findings showing that social dominance was closely associated with the dopaminergic reward system.

Thus, the 5-HT and dopamine systems is modulated by the hierarchical position of an individual. Reversely, the blood level of 5-HT appears to affect one's social status as well. Although dopamine was shown to act in the striatum, it is not clear whether similar change is observed in the other brain regions that expresses D2R and is reported to be engaged in dominance perception. Also, the primary site of action of 5-HT has not been determined in these studies.

### Oxytocin system

In mammals, including humans, oxytocin plays an important role in the regulation of complex social cognition and social behaviors such as attachment, social recognition, social exploration, aggression, and anxiety (for a review, see Meyer-Lindenberg et al., 2011; Kumsta and Heinrichs, 2013). Several non-human studies have demonstrated the influence of oxytocin on the formation and maintenance of a social hierarchy. According to their social hierarchy, dominant female rhesus macaque monkeys had higher serum oxytocin levels than those of subordinate monkeys (Michopoulos et al., 2011). Similarly, the mRNA expression of oxytocin receptor-related genes in the medial nucleus of the amygdala was lower in subordinate rats than in dominant rats (Timmer et al., 2011). However, the precise functional role of oxytocin in the perception and learning of social dominance remains unclear.

### NPBWR1 (GPR7) system

In contrast to monoaminergic system and oxytocin which distribute in wide areas of the brain, Neuropeptide B (NPB) and Neuropeptide W (NPW) system show limited localization (O'Dowd et al., 1995; Lee et al., 1999; Brezillon et al., 2003; Tanaka et al., 2003). NPBWR1 (or GPR7) is G_*i*_-protein-coupled receptor and is highly conserved in specific region in the brain of humans and rodents. *NPBWR1* mRNA has been localized to discrete brain regions including the hypothalamus, hippocampus, ventral tegmental area, and central nucleus of the amygdala in rodents (Lee et al., 1999; Tanaka et al., 2003), and the amygdala and hippocampus in humans (Brezillon et al., 2003). In behavioral tests, *Npbwr1*^−/−^ mice exhibited a shorter latency to initial physical contact and longer contact and chase times with the intruder during a resident–intruder test compared with *Npbwr1*^+/+^ mice, indicating decreased social fear (Nagata-Kuroiwa et al., 2011). However, because there were no significant differences between *Npbwr1*^−/−^ and *Npbwr1*^+/+^ mice in an open field test or an elevated plus maze test, this type of compulsive behavior toward the intruder does not seem to be indicative of an increase in general anxiety. Instead, this suggests that these changes were specific to the fear or anxiety experienced in a social context.

Watanabe et al. (2012) investigated behavioral differences during human social interactions and the relationships with *NPBWR1* gene variants. In humans, the *NPBWR1* gene may express a single nucleotide polymorphism (SNP), either 404AA or 404AT, at the site where this molecule binds to adenylate cyclase and subsequently regulates the function of this receptor. When human *404A* or *404T* genes were transfected into a HEK293A cell line, the *404T* gene was associated with lower levels of cAMP release compared with the *404A* gene, which indicates that the *404T* gene impaired receptor function. Because *Npbwr1*^−/−^ mice exhibited abnormal behaviors during social interactions (Nagata-Kuroiwa et al., 2011), it was hypothesized that a human with the *404AT* gene would be less sensitive to social context cues such as facial expressions.

Watanabe et al. (2012) presented pictures of four types of facial expression to their participants and asked them to evaluate their emotions during the presentation (see Section Facial Expression and Dominance). There was a significant difference between the genotypes during the evaluation of dominance such that the 404AT group felt less submissive during the presentation of an angry face than did the 404AA group. This suggests that individual differences in the SNP of NPBWR1 influence the perception of dominance, especially when participants observe overpowering stimuli, such as angry faces. Because *NPBWR1* mRNA expression occurs in limited areas, particularly in the amygdala in humans (Brezillon et al., 2003), this finding also supports the involvement of the amygdala in the perception of dominance during human interactions. However, the role of the Neuropeptide B/W system in the formation and maintenance of social hierarchies has yet to be directly validated.

## Discussion and conclusion

Psychological studies in the fields of personality and emotion have consistently demonstrated that the concept of dominance is a basic and indispensable factor that is inherent in interpersonal communication. Recent studies using clinical lesion cases, structural MRI, fMRI, and PET scans, and neuronal recording in animal models have characterized the neural substrates that support the perception, learning, and formation of social dominance and social hierarchies.

**Figure 2:**
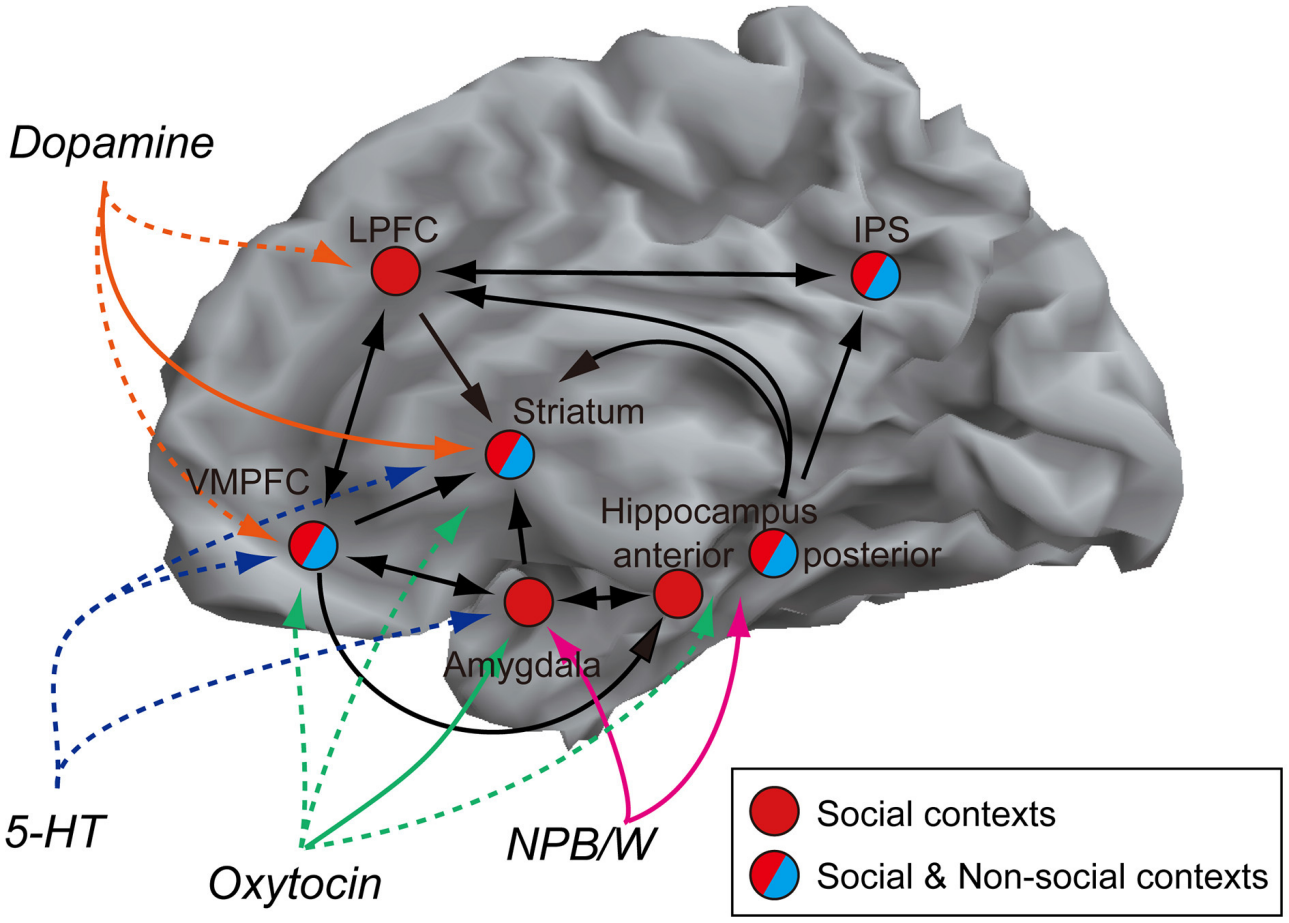
**Network model of social dominance.** Regions that have been reported to be involved in the perception of social dominance are shown. The black lines and arrows indicate possible direct connections between regions based on the anatomical studies (Clower et al., 2001; Freese and Amaral, 2009; Haber and Knutson, 2010; Yeterian et al., 2012). In terms of transmitters, the colored solid lines indicate target regions with scientific reports for hierarchy (Grant et al., 1998; Morgan et al., 2002; Brezillon et al., 2003; Timmer et al., 2011). The colored broken lines indicate possible regions that these transmitters could have effects (Passchier et al., 2000; Landgraf and Neumann, 2004; Hurd and Hall, 2005), but no scientific report in terms of social dominance and hierarchy.

In terms of perception and learning, no specific brain regions have been found to represent social dominance independently. Rather, the perception and learning of social dominance can be attributed to the integrated activity of various networks, which include the amygdala, striatum, hippocampus, IPS, VMPFC, and LPFC. Each region plays a different role that is specifically related to dominance signals (Table 1). We summarized the network that includes regions described in this article in reference to their anatomical connections (Figure 2) (Clower et al., 2001; Freese and Amaral, 2009; Haber and Knutson, 2010; Yeterian et al., 2012).

In this network, which part is a key component for the perception of dominance? It is hard to pinpoint, however, we suppose that the origin of the perception of dominance is a phylogenetically primitive part of the brain, because at the age of 15 months, children could already infer social ranking based on their own previous experiences (Mascaro and Csibra, 2012). In fact, even fish can infer social ranking (Grosenick et al., 2007). The amygdala is involved in the perception (Emery et al., 2001; Machado and Bachevalier, 2006; Zink et al., 2008) and learning (Kumaran et al., 2012) of social dominance and can influence the formation and maintenance of a social hierarchy (Rosvold et al., 1954; Noonan et al., 2014). Based on the available data, it is reasonable to assume that the amygdala is the primary brain region that supports the perception of dominance, because the majority of, if not all, studies have found that social hierarchy learning dynamics are represented in this region. The amygdala has afferent connections with hippocampus, striatum and VMPFC (Freese and Amaral, 2009). Thus, it is possible that the information of social rank (dominance) of a person is sent to these regions in which the knowledge and value associated with him/her is modulated (Phelps, 2004; Hampton et al., 2007; Stuber et al., 2011; Watanabe et al., 2013). The striatum receives afferent connection from the amygdala but no direct efferent fibers to the amygdala are reported, which indicates the possibility that social dominance information is sent to the striatum from the amygdala. The striatal activation is sensitive to pictures which represent similar social status as the participant's subjective one (Ly et al., 2011). Such representation of subjective value might reflect the modulation by the input from the amygdala. On the other hand, VMPFC has reciprocal innervation with the amygdala. It is thought that the amygdala supports the value calculation in VMPFC (Hampton et al., 2007) and conversely VMPFC regulates amygdala activity (Phelps et al., 2004; Cho et al., 2013); therefore value representation related to social dominance may also be modified by the amygdala-VMPFC interaction. Compared to a strong connectivity between the amygdala and VMPFC, the IPS projection from the amygdala seems weak (Freese and Amaral, 2009), but the IPS has connections with the hippocampus (anterior and posterior) (Clower et al., 2001), therefore both social and non-social dominance information might be sent from the hippocampus to the IPS. Such rank information could be processed as information of magnitude in this region.

The other area that is only associated with social dominance is the LPFC. The specificity of social context is consistent with recent reports of the specific engagement of the LPFC to socially normative behavior (Spitzer et al., 2007; Ruff et al., 2013). Because the normative behavior is influenced by social status (Cummins, 2000), it is possible that the LPFC may integrate social hierarchical information from IPS, hippocampus and VMPFC where information from the amygdala is processed, and support the execution of adaptive behavior based on the hierarchical relationship.

Furthermore, as summarized in Table 2, several neurotransmitters such as 5-HT, dopamine, oxytocin and NPB/W may modify activities of these networks. While the target site of 5-HT for dominance perception has not been identified so far, in terms of the dopamine system, the studies are focused on the striatum, and the expression level of D2R in the striatum is affected by an individual's social status in both monkey and human (Grant et al., 1998; Morgan et al., 2002; Martinez et al., 2010). A localized influence of oxytocin in relation to social rank was also shown in rats, specifically that mRNA expression of oxytocin receptor-related genes in the medial nucleus of the amygdala is affected by their status (Timmer et al., 2011). In addition, NPBWR1 is predominantly expressed in the amygdala and hippocampus (Brezillon et al., 2003) and plays a role in the perception of dominance in human (Solid colored lines in Figure 2).

Work in the hierarchy formation started early in the study of human psychology and animal experimentation, however research on neural substrates of dominance perception and hierarchy formation has just begun. Combinations of molecular and brain imaging technologies will advance the understanding of how these neural networks operate and how neurotransmitters modify activities in each region listed in this article in the context of dominance perception and hierarchy formation.

### Conflict of interest statement

The Guest Associate Editor Sonoko Ogawa declares that, despite being affiliated to the same institution as author Miyuki Yamamoto, the review process was handled objectively and no conflict of interest exists. The authors declare that the research was conducted in the absence of any commercial or financial relationships that could be construed as a potential conflict of interest.
